# Positive expression of basic transcription factor 3 predicts poor survival of colorectal cancer patients: possible mechanisms involved

**DOI:** 10.1038/s41419-019-1747-2

**Published:** 2019-07-01

**Authors:** Qi Liu, Junjie Wu, Tailiang Lu, Zhixue Fang, Zixuan Huang, Shanzheng Lu, Chen Dai, Mengqian Li

**Affiliations:** Department of General Surgery, People’s Hospital of Hunan Province, First Affiliated Hospital of Hunan Normal University, Changsha, Hunan Province China

**Keywords:** Predictive markers, Predictive markers

## Abstract

Basic transcription factor 3 (BTF3) is associated with the development of several cancers. The aim of our study was to elucidate the role of BTF3 in colorectal cancer (CRC) tissues. CRC tissues or their paired adjacent noncancerous (ANCT) tissues were obtained from 90 patients who underwent operations in our hospital from November 2011 to December 2016, and then we implemented a gene microarray assay for detecting significant changes in gene expression and confirmed expression in tissues using immunohistochemistry and real-time PCR. We transfected or injected the silencing BTF3 (BTF3-siRNA) plasmid into cells and nude mice, and measured the tumorigenicity of CRC cells with flow cytometry and studied the expression level of BTF3 downstream genes (MAD2L2, MCM3 and PLK1) in CRC cells. BTF3 expression level was not only significantly higher in CRC tissue than in ANCT tissue (2.61 ± 0.07 vs 1.90 ± 0.03, *P* < 0.001) but BTF3-siRNA decreased tumor formation in a nude mice model. Furthermore, based on the data of gene microarray analysis, MAD2L2, MCM3 and PLK1 were detected as the downstream target genes of BTF3 and their expressions were positive related with BTF3 expression. Also, through transfecting BTF3-siRNA into HCT116 cells, we found that BTF3-siRNA could decrease cell viability and induced cell apoptosis and blocking the cell cycle. In conclusion, BTF3 is positively related to CRC and BTF3-siRNA attenuated the tumorigenicity of colorectal cancer cells via MAD2L2, MCM3 and PLK1 activity reduction.

## Introduction

One of the most predominant malignancies in China is colorectal cancer (CRC) whose incidence in the Chinese population continues to grow^1^. The metastases generated at an advanced stage of CRC are the main mortality cause. It is likely that the development of sophisticated screening techniques to detect CRC at a very early stage will greatly change the mortality rate^2,3^. At present, an invasive screening colonoscopy method is considered to be the golden standard for diagnosis, but the quality of a screening colonoscopy relays on the technician level, training and quality control^4^, thus, colonoscopy capacity has a limit for its widespread use as a primary screening test. It would be ideal, to develop an alternative modality based on blood biomarkers as the first line non-invasive screening test.

The most common known related genes in CRC include APC, CTNNB1, KRAS, BRAF, SMAD4, TGFBR2, TP53, PIK3CA, ARID1A, SOX9, FAM123B, and ERBB2, which appear to promote colorectal tumorigenesis by perturbing the function of key signaling pathways, including the WNT-β-catenin, EGF-MAPK, PI3K and TGF-β signaling pathways, or by affecting and regulating core functions of cells such as DNA repair and proliferation^5^. But actually, in clinical practice few biomarkers are available for non-invasive screening from blood samples or fecal immunochemical tests and there is a growing expectation on the development of diagnostic tests based on more sensitive and specific molecular markers and those tests may provide a breakthrough to the limitations of current screening tests for CRC.

Basic transcription factor 3 (BTF3) was first isolated from HeLa cells, purified and proven to have important actions in accurate transcription initiation from the adenovirus-2 major late promoter (Ad2MLP) and various other promoters of class B RNA polymerases^6^. Two complementary DNAs have been cloned based on nucleotide sequences of BTF3 peptides, namely (1) BTF3a, a protein having many features characteristic of purified BTF3 and (2) BTF3b which is protein with a shorter base sequence. It has been demonstrated that BTF3b lacks the first 44 residues present in BTF3a. Surprisingly it cannot initiate transcription even though it readily binds to RNA polymerase II^7^. Increasing evidence has shown that, as a transcription factor, BTF3 is associated with the development of several types of cancers. BTF3 has also been shown to be involved in the stimulation of proliferation of cells, reducing of cell cycle regulation and gastric cancer cell apoptosis^8^. Furthermore, BTF3 is closely related to the development of prostate cancer^9^. Additionally, overexpression of BTF3 in pancreatic cancer cells^10^, where it functions to regulate transcription per se as opposed to direct modulation of apoptosis through actions on genes associated with cancer including ABL2, ATM, EPHB2 and HPSE2. Furthermore, it is known that MADCAM1, KRAG, RRAS2, NF-κB, MRVI1 are upregulated among others^10^. Interestingly, it has also been reported that BTF3 is positively correlated with colon cancer detected by clinicopathology^11^. We previously reported that BTF3 is involved in the development/progression of gastric cancer. However, the underlying mechanisms through which BTF3 regulates the development of CRC have not been fully elucidated.

On the other hand, previous research into colon cancer reported that the overexpression of mitotic arrest deficient 2‐like protein 2 (MAD2L2), a cancer suppressor protein that can inhibit CRC cell proliferation, migration and the ability of cells to form clones by triggering the degradation of nuclear receptor coactivator 3 (NCOA3)^12^. Overexpression of the mini-chromosome maintenance 3 (MCM3) gene has been reported in colon and other cancers, and has been implicated in a number of forms of carcinogenesis in humans in maintaining cancer cell growth^13^. However, it has been documented that adenomatous polyposis coli (APC) germline mutations cause aneuploidy and are responsible for familial adenomatous polyposis (FAP), while higher polo-like kinase 1 (PLK1) gene expression can increase the survival for colon cancer patient with a truncated APC.

As far as we are aware, our research is the first to study the role of BTF3 in the development of CRC, especially for rectal adenocarcinoma by the use of in vitro and in vivo animal models in trying to identify the downstream genes regulated by BTF3 and their probable mechanisms and relationships to known colon suppressor or inducer factors.

## Materials and methods

### Tissue samples obtained from patients

Human CRC (*n* *=* 90) and paired ANCT tissues (*n* = 90), median age 63 years; (range, 35–83) who underwent resection of the colorectum between November 2011 and December 2016 (each sample punching four cores, 0.6 mm in diameter per core) at the first affiliated hospital of Nanjing medical university. All patients were recruited following informed consent and the follow-up was carried out till December 2016. This study was approved by the medical ethics committee of the People’s Hospital of Hunan Province. The grouping of patients were depended on their histopathological tumor node metastasis (TNM) staging criteria, namely: patients with stage I or stage II, and patients with stage III or IV disease.

### Treatment of samples

Samples from resection partly were immersed in paraformaldehyde solution for 24 hours before being embedded in paraffin for histological analysis. Some samples were exposed to RNAlater™ stabilization solution (Ambion Ltd., Huntingdon, UK) for later microarrays. Alternatively, some samples were immersed in liquid nitrogen for rapid freezing and stored at −80 °C until their protein content was analyzed. The Human Ethics Committee of our hospital approved the research and consent was provided by all of the participants in the study.

### Immunohistochemical analysis

Samples were embedded in paraffin and 5-μm sections prepared for analysis by immunohistochemical protocols^14^. Briefly, sections were dewaxed, rehydrated in ethanol solutions of appropriately graded concentrations before being immersed in a 3% H_2_O_2_ solution in a light-free environment for 10 min at ambient room temperature to block the activity of endogenous peroxidase prior to antigen retrieval. Next, samples were blocked with serum homologous with the secondary antibody for 20 min at 37 °C. The tissue slices were incubated with primary rabbit anti-BTF3 (a/b) antibody (1:1000; Cell Signal Technology, US) overnight at a temperature of 4 °C. Sections were washed with phosphate buffer saline for 15 min, before incubation with anti-rabbit antibody that was conjugated with horseradish peroxidase (1:1000; Santa Cruz Biotech Company, US) for 40 min at 37 °C. Then the samples were reacted with 3, 3′-diaminobenzidine and also stained with Mayer’s hematoxylin solution.

### Cell culture

HCT116 cells were incubated in a Dulbecco modified eagle medium that was supplemented with 10% fetal bovine serum (Thermo Fisher Scientific Inc., US) in a 5% CO_2_ atmosphere at 37 °C.

### Lentivirus encoding BTF3-siRNA transfection of the human colon cancer cell line (HCT116)

Gene transfer of HCT116 cells was performed following a previously published method^15^. Briefly, after 5 days of culture, the cells were transduced with a lentivirus encoding BTF3-RNAi (sense 1: GCCGAAGAAGCCTGGGAATCA, antisense 1: TGATTCCCAGGCTTCTTCGGC; sense 2: CAAACAATCTGTGGATGGA antisense 2: TCCATCCACAGATTGTTTG sense 3: CAGTGATCCACTTTAACAA antisense 3: TTGTTAAAGTGGATCACTG and negative RNAi (sense: TTCTCCGAACGTGTCACGT, antisense: ACGTGACACGTTCGGAGAA) (all from Genechem, Shanghai, China), final we found the pair of sense 1 and antisense 1 was the most strong inhibition of BTF3 expression. The serum concentration, incubation time and virus concentration was determined to establish the optimum conditions for HCT116 lentivirus gene transfer. After conducting preliminary experiments, HCT116 cells were transduced with 10 MOI LV-BTF3-siRNA or LV-negative-siRNA for 16 h in 20% serum medium. After transduction for 72 h, the cells were collected for the determinations of inhibition with BTF3-RNAis, and found sense 1and antisense 1 was a best siRNA of BTF3 for further experiments.

### Assay to determine viability of cells

To determine the viability of cells, the methyl thiazolyl tetrazolium (MTT) assay was carried out following the manufacturer’s instruction (Sigma-Aldrich Chemie Gmbh Munich, Germany). In brief, the cancer cell were plated onto 96-well plates for indicated treatments, 20 µL of a 5 mg/mL MTT solution (Sigma) was applied to each well after teatments. After 4 h incubation, the solution was replaced with 150 µL of DMSO. A wavelength of 490 nm was employed to measure the absorbance of each well (Universal Microplate Reader, Bio-Tek Instruments); wells that did not contain cells were used as the control blanks. Absorbance readings of each treated group were normalized to the control values.

### Analysis with Annexin-V-fluorescein isothiocyanate (FITC)

The degree of apoptosis of transfected cells was assessed with an Annexin-V-FITC kit (BD Biosciences, New York, USA) following the manufacturers protocol. After 48 h transfection, HCT116 cells were collected, rinsed in PBS buffer and suspended in 1 × binding buffer (1 × 10^6^ cells/mL). Then, 5 µL Annexin-V-FITC was applied and the cells incubated for 15 min at ambient room temperature. Then cell analysis was carried out using flow cytometry BD FACSDiva.software v6.1.3 and BD CellQuest. Pro (BD Biosciences, New York, USA).

### Cell cycle assays

HCT116 cells were counted (1 × 10^5^ cells in each well) using flow cytometry and seeded into six-well culture plates for subsequent analysis of the cell cycle. The medium was substituted after 24 h with Dulbecco modified eagle medium containing a supplement of 1% FBS to make the cells dormant after 24 h. And then indicated treatments were applied to the HCT116 cells for another 24 h and then they were exposed to 80% ethanol and stored in a freezer for 2 h to complete fixation. The plates containing the HCT116 cells were then carefully positioned in an ice bath and 0.25% Triton X-100 applied to each well for a total of 5 min. Cells were resuspended in 300 mL of PBS containing propidium iodide (40 mg/mL) and RNase (0.1 mg/mL) and then incubated for 20 min under light-free conditions at room temperature. Finally, analysis of the cell cycle was carried out, ≥10,000 cells were assessed with a FACScan flow cytometer (Becton Dickinson, Mountain View, CA, USA) and FlowJo ver. 7.1.0 (Tree Star, Ashland, OR, USA).

### Profiling gene expression using microarrays and bioinformatic analysis

HCT116 cells were placed in a culture flask (25-cm^2^) in FBS-free Dulbecco modified eagle medium in an atmosphere containing 5% CO_2_ at 37 °C for 24 h. Negative controls or lentivirus that encoded BTF3-siRNA were transfected into HCT116 cells, (incubation time 48 h) before collection of cells for further studies. Both tissue and HCT 116 cells were analyzed microarray and expression of genes profiling with GeneChip® PrimeView™ Human Gene Expression Array (Affymetrix, US), which included a library of gene probes (circa 20,000 *Homo sapiens* probes that were annotated). All analyses were carried out 3 times and a *P*-value cutoff of 0.05 and a log2 fold-value change of ≥1 used as the filter to identified the genes that were differentially expressed. The levels of genes that were differentially expressed were converted into *Z*-scores before hierarchical clustering according to average linkages and Euclidean distances. Subsequently, enrichment analysis of all differentially expressed gene sets was carried out in order to identify any pathways that were significantly enriched. Downregulated or upregulated genes (1-fold) are illustrated as heat maps for selected pathways that were enriched.

### Western blotting

Lentivirus encoding negative control-siRNA or BTF3-siRNA were transfected into HCT116 cells for 48 h and then the cells were collected in lysis buffer. The total protein level was measured using BCA reagent (Thermo-Fisher, US) and the expression of various proteins by western blot analysis^16^. A 10% SDS-PAGE gel was used to distinguish proteins before transfer to a PVDF membrane and processed for 60 min at a voltage of 100. Next, the PVDF membrane was incubated in TBS/T: composition 150 mM NaCl, 20 mM Tris-HCl, 5% non-fat milk, 0.1% Tween-20 at pH 7.6 and equilibrated for 2 h at ambient room temperature. The specific primary antibodies were including rabbit anti-MAD2LC (1:1000; Cell Signaling Technology, California, USA), rabbit anti-MCM3 (1:1000; Cell Signaling Technology, California, USA), rabbit anti-PLK1 (1:1000; Cell Signaling Technology, US), and mouse anti-GAPDH (1:2000; Santa Cruz, US), were serially diluted in TBS/T buffer (composition: 150 mM NaCl, 50 mM Tris-HCl, 0.1% Tween-20) at pH 7.4; the membrane was maintained at 4 °C overnight to permit thorough incubation. The appropriate secondary antibodies that were conjugated with horseradish peroxidase were incubated with the membrane for 1 h at ambient room temperature. Signal detection was performed with an ECL reagent (Amersham Biosciences, Piscataway, NJ, USA).

### Xenograft model of nude mouse tumors

Eighteen female BALB/c nude mice (15–18 g, 4 weeks old) were purchased from SLAC branch of Shanghai lingchang biotechnology Co. LTD and the experiments approved by the Hunan Medical University ethics committee. Experimental animals were housed in a pathogen free environment: Steam, under high pressure, was used to sterilize chow and the drinking water was sterilized by irradiation with cobalt-60. The experimental mice were exposed to a 12 h light/dark cycle at a temperature of 24 °C ± 1 °C and a humidity of 55% ± 5%. The experimental animals were subcutaneously inoculated with HCT116 cells (1 × 10^7^) suspended in PBS (200 µL). When the tumor size reached ~10 mm in length, the mice were randomized into three groups (6 mice/group) to receive 15 µL PBS only (Control group), 15 µL LV-N-siRNA (N-siRNA group) and LV- BTF3 siRNA (BTF3-siRNA group) injected into the xenograft tumor in a multisite injection manner. Subcutaneous tumors were detected in all 18 experimental animals. The treatments were repeated at 4-day intervals and the sizes of the developing tumors were measured using a digital caliper. The volume of a tumor was determined using the equation: tumor volume (mm^3^) = [tumor length (mm) × tumor width (mm) × 2]/2. After 16 days the mice were humanely killed by cervical dislocation and individual tumors excised to determine their weights.

### Statistical analysis

To determine the relationship between clinicopathological factors and the expression of BTF3 pearson correlation methodology was employed. Kaplan–Meier curve and log-rank tests were employed to determine statistically the univariate biochemical recurrence-free survival of mice. To calculate multivariate and univariate hazard ratios for the studied variables we used a Cox proportional hazard regression model. The data are presented as means ± standard deviations (SD). In vitro differences between the experimental groups when data was normally distributed were examined using a Student’s *t*-test, with *P* < 0.05 being deemed to be statistically significant. Statistical analyses were carried out using SPSS for Windows (ver. 13, SPSS Inc., US).

## Results

### Clinicopathological features of colorectal cancer patients and the associated expression of BTF3

**Fig. 1:**
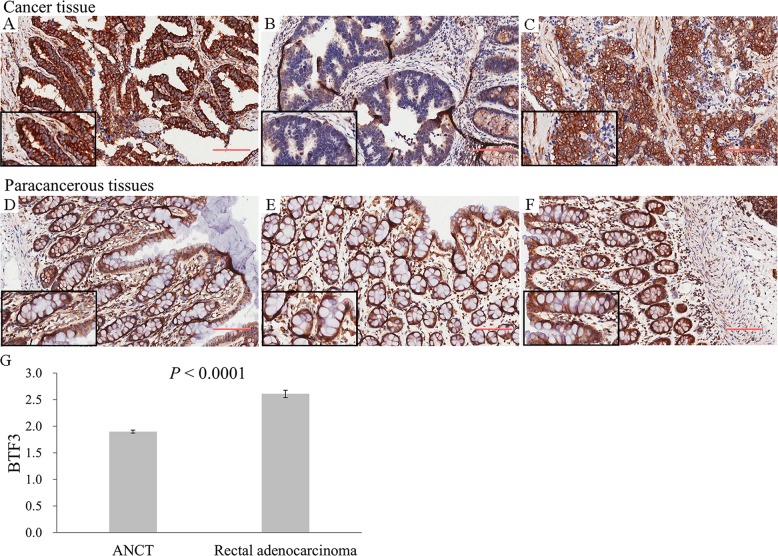
The inhibitory effects of BTF3 protein expression detected by IHC in clinical tissue array. Scale bar, 200 μm

**Fig. 2:**
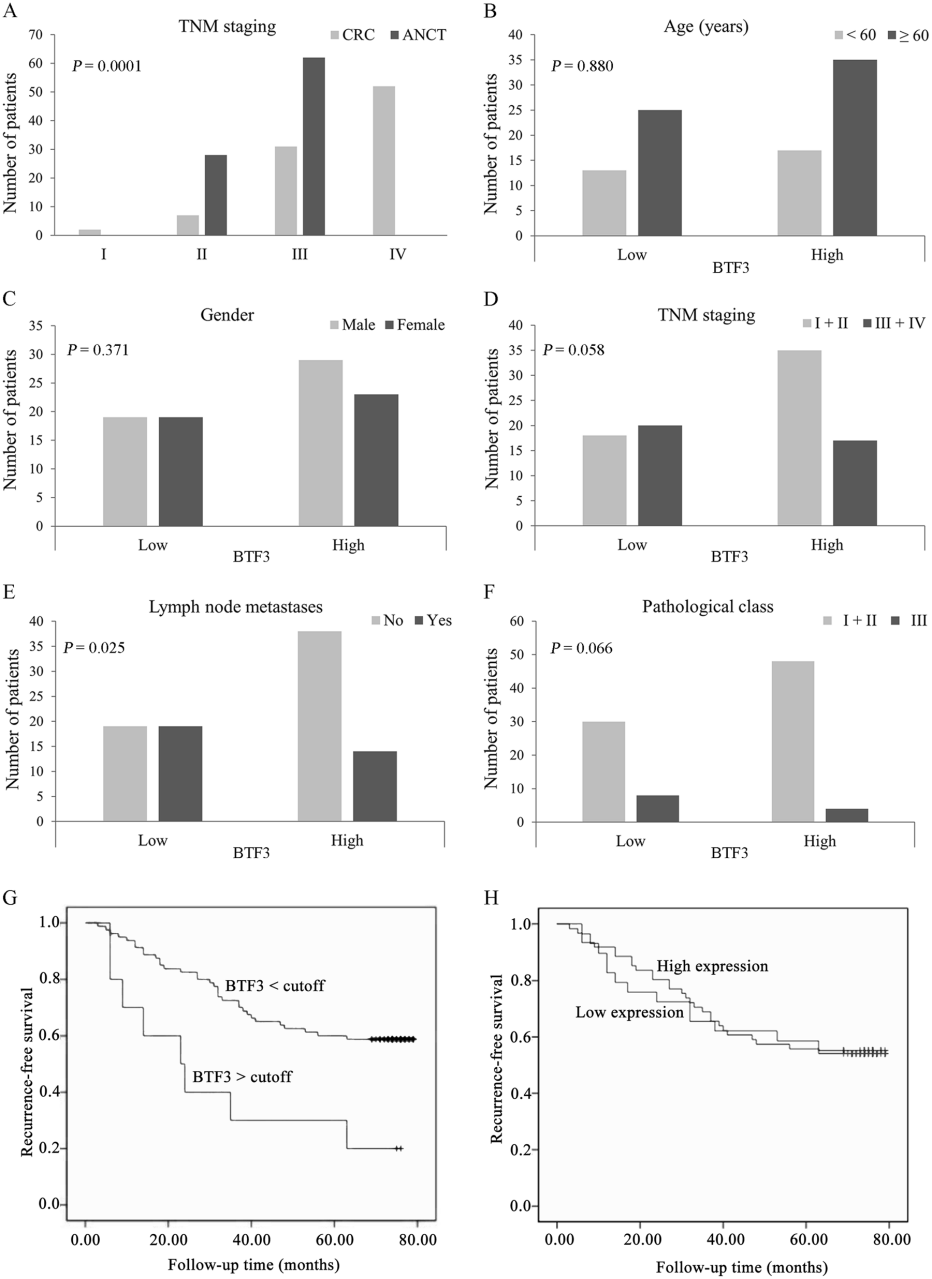
**a**–**f** Correlation of BTF3 expression with the clinicopathological characteristics of CRC patients. **a** The number of patients in different TNM stage; **b** the number of patients with higher BTF3 or lower BTF3 expression in two age group; **c** the number of patients with higher BTF3 or lower BTF3 expression in gender groups; **d** the number of patients with higher BTF3 or lower BTF3 expression in TNM group; **e** the number of patients with higher BTF3 or lower BTF3 expression in lymph node metastases tissues; **f** The number of patients in different pathological three class; **g**–**h** Kaplan–Meier survival curves of patients with different BTF3 expression levels. The overall survival after complete surgical resection was analyzed, and the cutoff was set at 3.0-fold BTF3 overexpression. Group 1, BTF3 expression higher than 3.0-fold (*n* = 38); Group 2, BTF3 expression below the cutoff value (*n* = 52)

BTF3 protein expression levels in 90 colorectal cancer patients were evaluated using IHC which clearly indicated a significantly different expression of BTF3 detected in tissue samples from ANCT (*n* = 90) and CRC (*n* = 90). Immunostaining revealed that cytosolic BTF3 was significantly higher expressed in the colonic epithelial cells (Figs. 1a–f). The relative immunoreactivity score of BTF3 in the ANCT samples was 1.90 ± 0.03 (derived from immunoreactive intensity measurements), significantly lower than those determined in CRC tissue (2.61 ± 0.07) (Fig. 1g, *P* < 0.001). Clearly, the expression of BTF3 was significantly greater in CRC tissue samples compared to levels in ANCT tissue (*P* < 0.001, Fig. 1g). These CRC tissues from the patients whose pathological characteristics showed that TNM (I + II) staging accounted for 58.9, 63.3% were in N0 of lymph node metastasis and M0 of distant metastasis, 86.7% patients were belonging to pathological class I + II, and there was a significant difference of BTF3 expression in different TNM staging between CRC and ANCT (Fig. 2a and Table 1).

**Table 1:** Characteristics of patients with CRC

	CRC (*n* = 90, %)
Age	63 years; (range, 35–83)
Gender	
Female	42 (46.7)
Male	48 (53.3)
TNM staging	
I + II	53 (58.9)
II + III	37 (41.1)
Lymph node metastasis (N1–N4)	
N0	57 (63.3)
N1–2	33 (36.7)
Distant metastasis	
M0	57 (63.3)
M1	33 (36.7)
Pathological class	
I + II	78 (86.7)
III	12 (13.3)

*CRC* colorectal cancer, *TNM* tumor node metastasis

Next, analyses of correlations between BTF3 expression (low and high expression) and a number of histopathological as well as clinical features of patients with CRC were conducted. However, there was no significant difference in TNM staging between group I + II and group III + IV whether with low or high expression of BTF3 (Fig. 2d, *P* = 0.058). Also there was no significant difference in age between over than 60 years old and less than 60 years old (Fig. 2b) or in gender (Fig. 2c), but the level of BTF3 expression was significantly higher in CRC with metastatic lymph nodes than in CRC without lymph node metastases tissue samples (Fig. 2e, *P* < 0.05). Additionally, the BTF3 IHC staining scores depending on the division of pathological class showed no significant difference (*P* = 0.066, Fig. 2f).

### Expression of BTF3 is an independent prognostic parameter indicating the survival times of CRC patients

A total of 39 patients died of cancer-related issues during 5-year following-up. The tissue samples taken from patients that overexpressed BTF3 significantly correlated to a reduction in their recurrence-free survival (log-rank test, *P* = 0.003) in follow-up times, which indicated that the aberrant higher expression of BTF3 was linked to a poor prognosis following Kaplan–Meier survival analysis (Fig. 2g), but there was no significant difference of recurrence-free survival between overexpressed BTF3 and lower expressed BTF3 in ANCT tissues (Fig. 2h). Furthermore, the risk factors of overall survival were analyzed in 90 patients (Table 2) using univariate and multivariate cox regression models. The significant prognostic variables for survival were the late stage of TNM (III-IV) (*P* = 0.038), lymph node metastases (*P* = 0.013), and the overexpression of BTF3 (*P* = 0.003) in an univariate regression model, and after using a multivariate analysis, we found only late stage of TNM (III-IV) and higher expression of BTF3 (over than cut-off values based on 3-fold overexpression of BTF3 in control) were poor indicators for recurrence-free survival.

**Table 2:** Prognostic risk factors analysis of overall survival in CRC patients

	Univariate analysis		Multivariate analysis	
	HR (95% CI)	*P*-value	HR (95% CI)	*P* value
Age	0.400 (0.213–0.806)	0.527		
Sex	1.278 (0.689–2.025)	0.258		
Tumor size (≤5 cm vs >5 cm)	0.848 (0.412–1.564)	0.357		
TNM staging (III-IV vs I-II)	2.288 (1.269–4.362)	0.038	2.594 (1.396–4.798)	0.003
Lymph node metastases	6.138 (3.214–10.051)	0.013		
Pathological class (III vs I-II)	4.908 (2.378–9.120)	0.086		
BTF3 expression less cutoff	9.046 (4.849–17.124)	0.003	0.367 (0.158–0.851)	0.020

*BTF3* basic transcription factor 3, *TNM* tumor node metastasis

### BTF3 expression correlated with CRC by using BTF3 silencing on the proliferation of HCT116 cells

**Fig. 3:**
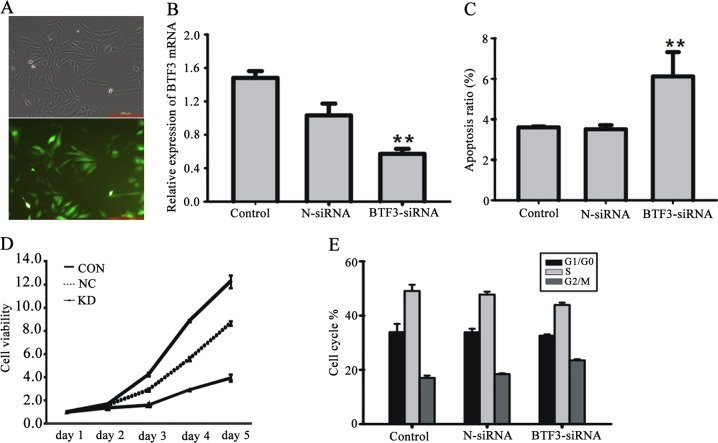
Effect of BTF-siRNA on HCT116 cell proliferation. (**a**) BTF3-siRNA was successfully transfected into HCT16 cells (scale bar, 300 µm) and (**b**) significantly reduced BTF3 expression, (**c**) enhanced the apoptosis rate and (**d**) reduced cell viablility. (**e**) BTF3 silencing led to blocked cell cycle. Values are expressed as mean ± S.E.M. (*n* = 3). ^**^P < 0.01, BTF3-siRNA *vs*. N-siRNA

To investigate more detailed the effects of BTF3 expression on the development of colon cancer, BTF3 expression was successfully silenced by LV-BTF3-siRNA transfected into HCT116 cells based on pre-screen results of siRNA sequence efficacy (Fig. 3a) and confirmed that LV-BTF3-siRNA successfully inhibited endogenous BTF3 expression in the cells (Fig. 3b). Compared with N-siRNA (negative control), BTF3-siRNA induced cell apoptosis (control 3.5% ± 0.2% *vs.* BTF3-siRNA 6.1% ± 1.2%, *P* < 0.01, Fig. 3c) and impaired the cell viability (Fig. 3d). Moreover, we found that BTF3 silencing also blocked the cell cycle of HCT116 cells in the G2/M phase (18.4% ± 0.3% *vs.* 23.5% ± 0.4%, *P* < 0.01) (Fig. 3e).

### Inhibitory effects of BTF3-siRNA on growth in a nude mouse model

**Fig. 4:**
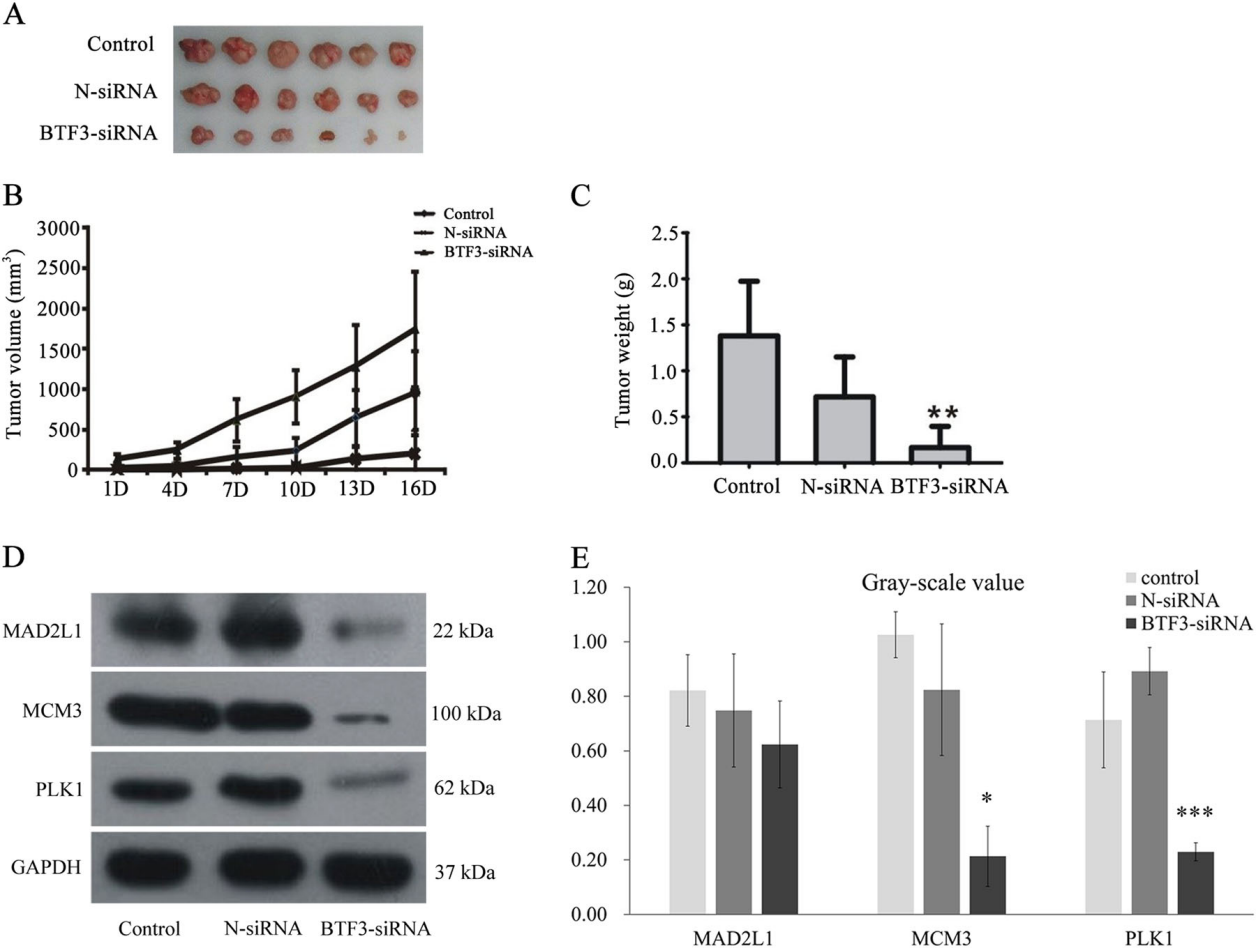
**a**–**c** Effect of BTF-siRNA on tumor growth in the nude mice model. **a** The presentative picture of tumor size. **b** The summary of tumor volume. **c** The summary of tumor weight. Values are expressed as mean ± S.E.M. (*n* = 9–10). ^**^P < 0.01 *vs*. N-siRNA. **d**–**e** Effect of BTF3-siRNA on MAD2L2, MCM3, and PLK1 protein expression. **d** the MAD2L2, MCM3, and PLK1 protein expression by western blot; **e** the relative amount expression of MAD2L2, MCM3, and PLK1 factors in HCT116 cells

Next, to confirm further that BTF3-siRNA can suppress tumor growth, we established a xenograft model using a lentivirus-mediated siRNA therapy protocol (Fig. 4a).

After treatment with lentivirus-mediated siRNA, the mean tumor volumes of the mice were measured, and in the blank control group, negative group (N-siRNA), and BTF3-siRNA group they were 143.38 ± 59.70 mm^3^, 72.60 ± 47.33 mm^3^, and 4.66 ± 8.25 mm^3^, respectively, with statistically significant differences between BTF3-siRNA and NC-siRNA groups (Fig. 4b). This effect of tumor suppression by BTF3-siRNA was aggravated with increasing time. On day 16, tumor volumes in the control (1,745.34 ± 712.90 mm^3^) and negative control (954.93 ± 517.12 mm^3^) groups rapidly increased when compared to the BTF3-siRNA group (202.60 ± 297.64 mm^3^, *P* < 0.05). Tumor weights in the negative group (0.72 ± 0.43 g) were significantly higher (*P* < 0.01) than in the BTF3-siRNA group (0.17 ± 0.23 g, Fig. 4c). Although, tumor weights in the negative group were inhibited compared with the blank control group (1.38 ± 0.59 g), the maximum inhibitory effect was induced by BTF3-siRNA (Fig. 4c).

### Differentially expressed genes in HCT116 cells treated by BTF3-siRNA detected via microarray assay

To determine the effect of genes regulated by BTF3-siRNA, microarray analyses of HCT116 cells treated by BTF3-siRNA or NC-siRNA were performed (https://www.ncbi.nlm.nih.gov/geo/query/acc.cgi?acc=GSE96755). The distribution of probes that were differentially expressed are shown as a volcano plot in Supplementary Fig. 1A. The dotted lines (red and green) are a representation of the cutoff, which is a measure of the fold change on the *x*-axis *vs* measurement of significance on the *y*-axis. The log_2_ scale expression signal values were plotted for all probes, excluding the control and flagged probes. The criteria adopted for the identification of the genes that were differentially expressed were: log2 ≥ 1 and *P* < 0.05. A total of 324 genes were upregulated and 649 downregulated in the BTF3-siRNA-transfection HCT116 groups compared with the control group transfected by N-siRNA (Supplementary Fig. 1B). Next we carried out functional enrichment analysis to determine the molecular functions and roles of the differentially expressed genes in regulatory signaling pathways that have previously been well characterized. The analysis showed that a number of signaling pathways were significantly enriched (score > 2.0, *P* *<* 0.05). The top 10 enriched pathways linked to BTF3 activity are documented in Supplementary Fig. 1C. Of these, cell cycle appeared to be the most enriched one (enrichment 18 genes, Supplementary Table 1). Since cell cycle regulation is widely accepted to occur during cancer development, it is highly probable that this signaling process has a vital function in BTF3-mediated development of colon cancer.

### BTF3 expression silencing regulation on MAD2L2, MCM3, and PLK1 expression

Based on the data of the microarray assay, we selected three genes involved in the cell cycle pathway, namely MAD2L2, MCM3, and PLK1. To explore the mechanism of BTF3 in colon cancer proliferation, expression levels of MAD2L2, MCM3, and PLK1 were detected. The results clearly demonstrate that BTF-siRNA significantly inhibits the expression of all these proteins (Figs. 4d-e).

## Discussion

Our investigations have revealed that BTF3 expression is upregulated in colorectal cancer tissue; BTF3 expression with 3-fold cutoff could be a good prognostic gene for CRC patients. KRAS, BRAF and PIK3CA were also presented as screen factors in colorectal cancers using their mutations^17^. It is noteworthy that epigenomic instability and microsatellite instability (MSI) including LINE-1 hypomethylation and the CpG island methylator phenotype are linked to mutations in oncogenes and prognosis in the clinic^18,19^. However, interrupted BTF3 protein expression could inhibit the viability and blocked the cell cycle of HCT116 cells. In the in vivo experiments, BTF3-siRNA inhibited the growth of rectal tumors, and in the microarray, we confirmed that BTF3 regulated the cell cycle related gene (MAD2L2, MCM3, and PLK1). It has been demonstrated that BTF3 is associated with the cell proliferation, a reduction in the cell cycle regulation and apoptosis in gastric, prostate and lung cancer^8,9,20^.

BTF3a is a transcription factor that plays a vital role in the transcription initiation by RNA polymerase through proximal promoter elements including CAAT and TATA box sequences^6,21–23^. Until now, the function of BTF3 was shown to be related to embryonic stem cells and tumor development by random mutagenesis screening of genes involved in development using the ROSA beta-geo retroviral genetrap vector^24^. BTF3 has also been shown to have actions in regulating the cell cycle and in apoptosis^25,26^. For example, altered BTF3 has been linked to the apoptosis of BL60 Burkitt’s lymphoma cells^27^. Studies of the downregulation of BTF3 revealed its role in inhibition of protein synthesis and transcription in K562 cells undergoing apoptosis^28^. In the latter study, BTF3 was found to be upregulated with CRC development, especially in lymph node metastases in the clinical research. Furthermore, microarray gene screen results showed that BTF3 related with many genes involved with cell growth and cell cycle.

We selected three genes be classified in mitotic spindle checkpoint genes related with cell cycle. It has been suggested that a defect in mitotic spindle checkpoint genes triggers aneuploidy in human cancer^29^. MAD2L2, a member of the mitotic arrest deficiency (MAD) gene family, is now known to be involved in the encoding of mitotic spindle checkpoint elements, delaying anaphase permitting all chromosomes to enter proper alignment. This process is brought about by inhibiting the activity of the anaphase-promoting complex. MAD2L2 has been shown to associate with a number of binding partners including enzymes responsible for repairing damage to DNA^30^. Therefore, MAD2L2 in involved the development of cancer. In fact, MAD2L2 has already been confirmed to be upregulated in CRC in a previous research^31^. MCM3 is believed to be the driving force behind replicative helicase, although it is noteworthy that it has no activity on the pre-replicative complex. Loading of MCM3 occurs only during G1 when the activity of cyclin-dependent kinase (CDK) is minimal and APC is active. Posttranslational modifications are crucial for the regulation of protein functions and their subsequent degradation during mitosis^32–34^. A critical role is by played PLK1 in the regulation of cytokinesis and mitosis, where it acts by phosphorylating target proteins. PLK1 also has vital actions on mitotic entry after damage has occurred to DNA^35,36^, centrosome separation^37^, in the stabilization of kinetochore-microtubule attachments^38,39^, removal of cohesin from sister chromatids^40,41^, and in the initiation of cytokinesis^42^. Taken together, according to our microarray findings, BTF3 seems to be association with MAD2L2, MCM3, and PLK1 through regulatory effects in different stages of mitosis, which affect proliferation and cell cycle of colorectal cancer cells. However, there is no direct physical protein interaction between this factors described, but using the Ingenuity pathway analysis (IPA) program (https://www.qiagenbioinformatics.com/products/ingenuity-pathway-analysis/) for a systematic bioinformatic analysis (Supplementary Fig. 2), the results showed that BTF3 might regulate the expression of MCM3 via interaction with p21 activated kinase 2 (PAK2) (BTF3 → PAK2 → MCM3)^43,44^ or Casein kinase 2, alpha 1 (CSNK2A1) (BTF3 → CSNK2A1 → MCM3)^45^ or RNA polymerase II subunit RPB2 (POLR2B) (BTF3 → POLR2B → MCM3)^7,46^ and BTF3 might interact with PLK1 via Centromere protein J (CENPJ) (BTF3 → CENPJ → PLK1);^47,48^ BTF3 might also interact with MAD2L2 via von Hippel-Lindau tumor suppressor (VHL) and kinesin family member 2 C (KIF2C) through MSD2L1 (BTF3 → VHL → KIF2C → MAD2L1 → MAD2L2)^49^. In addition, miRNA in silico analyses revealed that hsa-miR-92–3p targets BTF3 and MCM3 while hsa-miR-15–5p targets BTF3 and PLK1^49^.

## Conclusion

BTF3 was involved in the development of CRC and regulate MAD2L2, MCM3, and PLK1 in mitosis as well as interfered with overall survival in CRC patients. Hence, BTF3 might be a prognostic factor for CRC in clinical practice.

## Supplementary information

Supplementary materials
