# Supplementary material for: Positive expression of basic transcription factor 3 predicts poor survival of colorectal cancer patients: possible mechanisms involved
Source: Cell Death Dis. 2019 Jul 1;10(7):509. doi: 10.1038/s41419-019-1747-2 (PMC6603001; doi:10.1038/s41419-019-1747-2)
Supplement: Supplementary file 1 — Supplementary materials [file 41419_2019_1747_MOESM1_ESM.docx]

**Supplementary Table 1.** Enrichment pathways from the analysis of gene set enrichment

| Gene set names | Genes in overlap (k) | *P*-value |
| --- | --- | --- |
| Cell cycle | 18 | 1.01 × 10^−12^ |
| DNA replication | 10 | 9.9 × 10^−11^ |
| Ubiquitin-mediated proteolysis | 12 | 1.32 × 10^−6^ |
| Biocarta MCM pathway | 5 | 5.52 × 10^−6^ |
| Adipocytokine signaling pathway | 8 | 7.5 × 10^−6^ |
| Mismatch repair | 5 | 2.04 × 10^−5^ |
| Biocarta G2 pathway | 5 | 2.54 × 10^−5^ |
| Progesterone-mediated oocyte maturation | 8 | 4.75 × 10^−5^ |
| Valine, leucine, and isoleucine degradation | 6 | 4.91 × 10^−5^ |
| P53 signaling pathway | 7 | 8.11 × 10^−5^ |

MCM: mini-chromosome maintenance

**
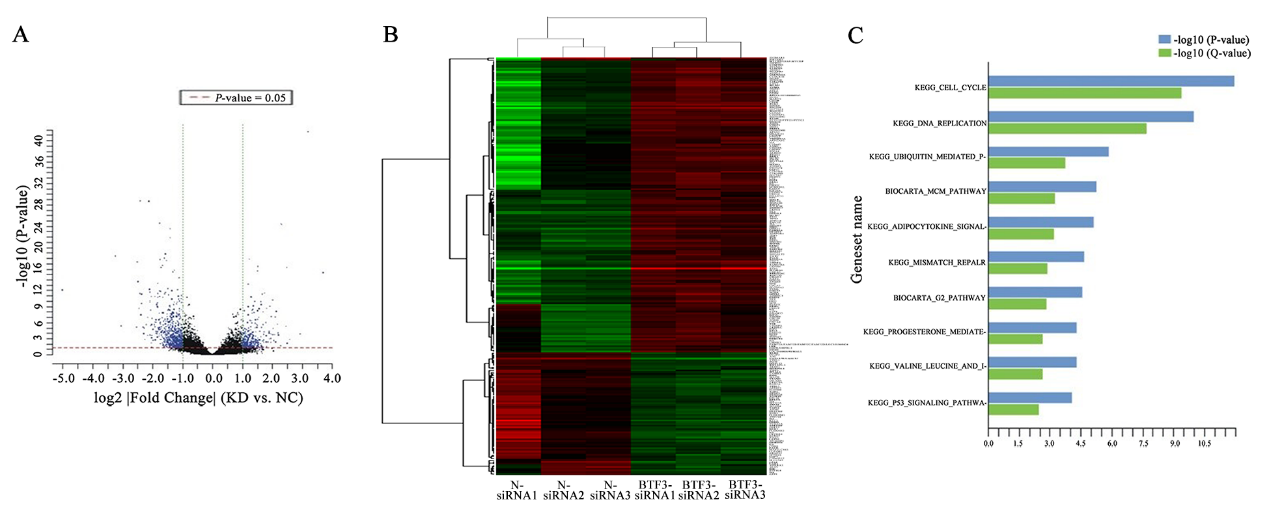
Supplementary Figure 1.** Effect of BTF3-siRNA on expression of different genes detected by microarray. A: The volcano plot of sample BTF3 versus N-siRNA. Standard selection criteria to identify differentially expressed genes are established at log_2_ |fold change| ≥ 1 and *P* value < 0.05 (Blue dots in figure). B: Clustering was performed to visualize the correlations among the replicates and varying sample conditions. Upregulated and downregulated genes are represented in red and green colors, respectively. A subset of differential genes was selected for clustering analysis. C: Top 10 enrichment pathway terms from gene set enrichment analysis on the y-axis versus a measure of significance (negative logarithm of the P value or Q value) on the x-axis. Q values were adjusted by FDR.

**
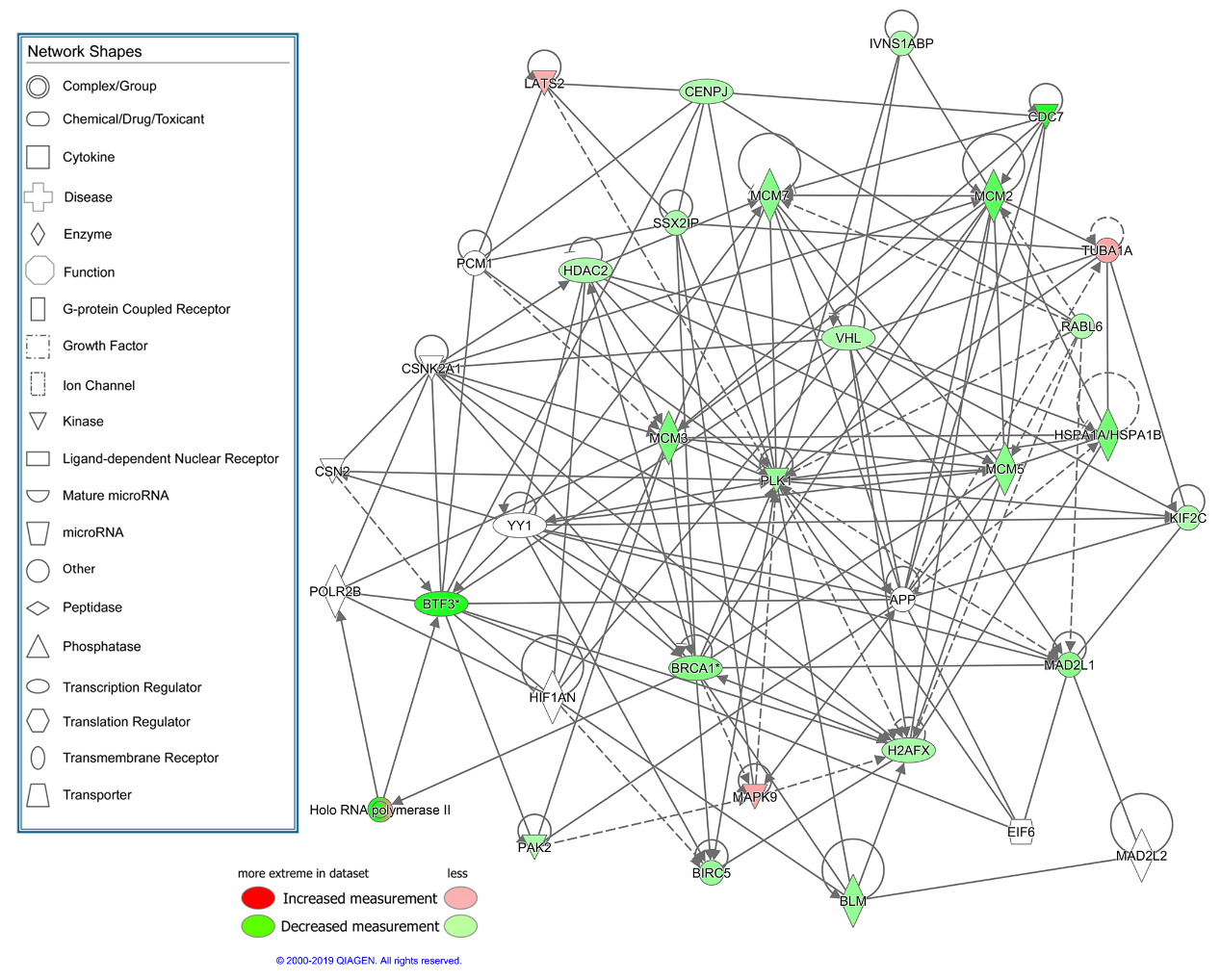
Supplementary Figure 2.** In silico analysis of protein interactions between BTF3 and MAD2L2, MCM3 and PLK1.
